# Comparison of Phoenix Sepsis Score and traditional scoring systems in predicting mortality in PICU patients with suspected infection

**DOI:** 10.1038/s41598-026-47381-1

**Published:** 2026-04-03

**Authors:** Dianwei Xing, Zijing Xiao, Baiming Liu, Jingjing Xu, Yufeng Liang

**Affiliations:** Department of Pediatric Critical Care Medicine, Guangzhou Women and Children’s Medical Center, Guangzhou Medical University, Guangzhou, 510623 Guangdong China

**Keywords:** Phoenix Sepsis Score, Mortality, Infection, Organ dysfunction, PICU, Diseases, Health care, Medical research, Risk factors

## Abstract

Sepsis is a major contributor to mortality in pediatric intensive care units (PICU). Timely and accurate risk stratification in children with sepsis is essential to guide clinical management. The objective of this study was to evaluate and compare the ability of the Phoenix Sepsis Score (PSS), Phoenix-8 score, systemic inflammatory response syndrome (SIRS) score, Pediatric Sequential Organ Failure Assessment (pSOFA), Pediatric Logistic Organ Dysfunction-2 (PELOD-2) score, and Pediatric Risk of Mortality III (PRISM III) score to predict in-hospital mortality among PICU patients with suspected infection in low- and middle-income countries. We conducted a prospective, single-center cohort study of children admitted to the PICU with suspected infection. The primary outcome was in-hospital mortality. We assessed scoring system discrimination by calculating the area under the receiver operating characteristic curve (AUC) and evaluated calibration using the Hosmer–Lemeshow test. Among 519 eligible children, 32 in-hospital deaths occurred (6.2%). The median time to death was 4 days (IQR, 1–9). For mortality prediction, the AUCs were 0.86 (95% CI, 0.77–0.95) for PSS, 0.89 (95% CI，0.82–0.95) for Phoenix-8, 0.73 (95% CI，0.63 –0.83) for SIRS, 0.87 (95% CI，0.79–0.94) for pSOFA, 0.90 (95% CI，0.85–0.96) for PELOD-2, and 0.89 (95% CI，0.82–0.97) for PRISM III. PSS exceeded SIRS and was comparable to pSOFA for in-hospital mortality prediction, while Phoenix-8, PELOD-2, and PRISM III showed higher AUCs. For the combined outcome of early death (within 72 h of PICU admission) or extracorporeal membrane oxygenation (ECMO) use, PSS showed similar performance to pSOFA, Phoenix-8, and PELOD-2. No significant miscalibration was observed across scoring systems. PSS demonstrated good discriminatory ability in identifying high-risk patients for mortality in the context of low- and middle-income countries. It is suitable for the early identification of adverse outcomes in PICU patients with infections.

## Introduction

Sepsis continues to be one of the top causes of death in children worldwide, with an estimated 25 million pediatric cases annually. The greatest burden is on children under five years old. Over 3 million children die from sepsis each year, especially in resource-limited settings^1–3^. Although antimicrobial treatments, organ support technologies, and intensive care have advanced, infection-related complications remain common in the PICU, particularly in patients who experience rapid deterioration and early multiple organ dysfunction. Timely recognition of sepsis and organ failure is key to improving survival rates and clinical outcomes for critically ill children^4^. However, accurately forecasting outcomes in pediatric sepsis remains a substantial clinical challenge.

Several scoring systems are commonly employed in clinical practice and research to assess prognosis in children with infection or sepsis. The International Pediatric Sepsis Consensus Conference (IPSCC) criteria have long provided a diagnostic framework for pediatric sepsis^5^. However, after the release of the Sepsis-3 definition in 2016^6^, there is still no pediatric-specific guidance for its application^7^. Although the pSOFA score helps quantify organ dysfunction in pediatric patients, it was adapted from the adult SOFA framework rather than being specifically designed for children^8^. In addition, the PELOD-2 and PRISM III scores are mainly used to assess disease severity and mortality risk^9,10^. However, these tools differ greatly in terms of their objectives, variables, and clinical settings, and some require many parameters, which can limit their use in low- and middle-income countries.

In 2024, the Pediatric Sepsis Definition Task Force of the Society of Critical Care Medicine issued updated diagnostic criteria for pediatric sepsis and septic shock and presented the PSS and its extended version, Phoenix-8^11,12^. PSS emphasizes organ dysfunction caused by infection. It is simple to apply and uses bedside data that clinicians can readily obtain in routine practice. The score is designed to support early recognition of sepsis and severe adverse outcomes in critically ill children. Early clinical studies evaluated PSS in emergency departments and in a subset of PICU cohorts. They showed that PSS may better predict mortality and other serious outcomes than some existing tools^13–17^. Yet, most validation studies were performed in high-income countries. As a result, the transportability of PSS across different healthcare systems is still unclear. This issue is particularly important in low- and middle-income countries, where sepsis care often faces limited resources, inadequate equipment, and persistent shortages of trained staff. Evidence from these settings is still scarce.

Therefore, this study aimed to use a single-center PICU cohort from low- and middle-income countries to systematically compare the ability of PSS, Phoenix-8, SIRS, pSOFA, PELOD-2, and PRISM III in predicting in-hospital adverse outcomes among children in the PICU with confirmed or suspected infection.

## Methods

### Study design

**Fig. 1:**
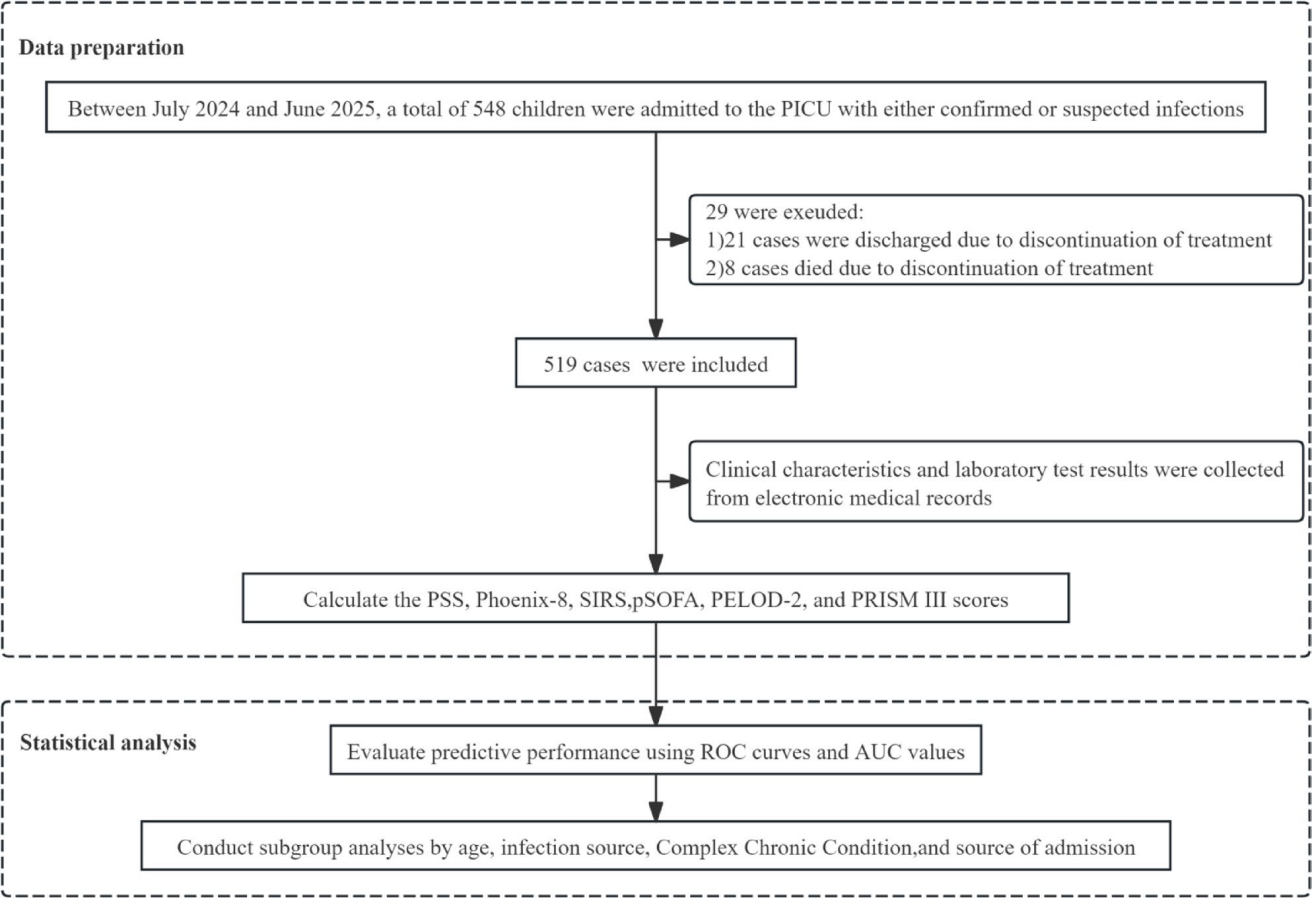
Flowchart of patient inclusion with confirmed or suspected infection.

This prospective, single-center cohort study was conducted between July 2024 and June 2025 in the PICU of a tertiary pediatric medical center in South China. The PICU is a regional referral center providing critical care for children with severe medical and surgical conditions. The study included all patients who were admitted to the PICU between July 2024 and June 2025 with confirmed or suspected infections. Patients were excluded based on the following criteria: (1) age ≤ 28 days or ≥ 18 years; (2) transfer to another PICU during their hospitalization; (3) death within two hours of PICU admission; (4) death following the withdrawal of life-sustaining treatment; and (5) discharge against medical advice upon request of their guardians. These exclusion criteria were applied to ensure complete and reliable ascertainment of in-hospital outcomes. For patients with multiple PICU admissions during the study period, readmission occurring more than 48 h after transfer to a general ward was considered a new episode, whereas admissions occurring within 48 h were treated as a single episode. We followed all enrolled patients until hospital discharge or death. Children who remained hospitalized at the end of the study period were censored and considered alive at the time of censoring. This was an observational study, and no study-specific diagnostic or therapeutic interventions were implemented. All data were collected in an anonymized manner, and each child was identified using a unique admission number. The Ethics Committee of Guangzhou Women and Children’s Medical Center, Guangzhou Medical University approved the study protocol (Approval No. 323A01). We conducted the study in accordance with the Declaration of Helsinki and its subsequent amendments and obtained written informed consent from all legal guardians. Figure 1 illustrates the patient enrollment process.

### Outcomes and definitions

In this study, the primary outcome was in-hospital mortality, which served to assess the potential for life-threatening organ dysfunction in patients with infection. The secondary outcome was a composite measure of early death or the need for ECMO support. Early death was defined as death within 72 h of PICU admission, as it is more closely linked to sepsis, which typically occurs within the first 24 h after PICU admission, compared to in-hospital mortality that may arise from complications occurring later in the hospitalization. Suspected infection was defined as receiving systemic antimicrobial therapy and undergoing microbiological testing within 24 h of PICU admission. This aligns with the standards established during the PSS process^11^. The diagnosis of central nervous system (CNS) infection was based on a comprehensive evaluation that included clinical neurological symptoms (such as altered consciousness or seizures), cerebrospinal fluid abnormalities, and imaging evidence. Cases meeting these criteria were categorized as CNS infections, excluding those with sepsis-associated encephalopathy (SAE) or other non-primary CNS disorders.

### Data collection

Demographic and clinical data were prospectively gathered, including sex, age at admission, diagnosis, source of admission (emergency department, general ward, or transfer from another hospital), mechanical ventilation use, PICU length of stay, and clinical outcome (survival or death). Comorbidities were recorded based on medical history and classified according to the presence of complex chronic conditions when applicable. The physiological and laboratory parameters needed to calculate the PSS, Phoenix-8, SIRS, pSOFA, PELOD-2, and Prism III scores were extracted from the electronic medical records. Data from the first 24 h after PICU admission were utilized for these calculations. If a variable was recorded multiple times on the same day, the most extreme value recorded that day was used for scoring, based on standard operating procedures. In this study, missing data were primarily concentrated in the D-dimer variable for the PSS score, with 179 missing cases(34.5%). According to the scoring system, unmeasured variables were assigned zero points^11^. SIRS was calculated according to the 2005 pediatric sepsis consensus criteria^5^. pSOFA was calculated using the Matics version^8^. To ensure data accuracy and consistency, one designated PICU staff member was responsible for data entry, and a second staff member independently supervised the process. A standardized electronic tool was developed to automatically generate each score after entry of the required parameters, and all entries were verified by senior clinicians.

### Statistical analysis

We performed all statistical analyses using R software (version 4.4.1). We summarized continuous variables as medians with ranges and reported categorical variables as frequencies and percentages. Before formal comparisons, we assessed data distribution for normality. For normally distributed variables, we expressed results as mean ± standard deviation and compared groups using t-test. For non-normally distributed variables, we reported medians with interquartile ranges and applied the nonparametric rank-sum test for group comparisons. We evaluated the discriminative performance of each scoring system for predicting survival using the area under the receiver operating characteristic curve (AUROC) with 95% confidence intervals. AUC values of 0.70–0.79 were considered acceptable, values ≥ 0.80 were considered good, and values ≥ 0.90 were considered excellent. We evaluated the calibration of the scoring systems using the Hosmer–Lemeshow goodness-of-fit test, including PSS, Phoenix-8, SIRS, pSOFA, PELOD-2, and PRISM III. We used the DeLong test to assess the statistical significance of the difference in AUC values. Although the traditional Hosmer–Lemeshow test commonly uses ten groups, several scoring systems in this study produced highly discrete predicted probabilities with frequent tied values. To address this issue, we therefore adopted an adaptive binning approach. For each scoring system, the number of groups was gradually reduced from g = 10 until a valid test statistic without binning warnings could be obtained. The resulting optimal g value was used for final reporting. P values were calculated based on the chi-square distribution. A P value greater than 0.05 was considered indicative of good agreement between predicted and observed values, whereas a P value less than 0.05 suggested poor calibration. We further performed subgroup analyses of the primary outcome according to baseline characteristics, including age group, infection source, comorbidity status, and admission source. All analyses applied two-sided tests, and we considered a P value < 0.05 statistically significant unless otherwise noted.

## Results

Between July 2024 and June 2025, we enrolled 519 children admitted to the PICU with confirmed or suspected infection. Table 1 summarizes their baseline demographic and clinical characteristics. Respiratory infection was the most common source, accounting for 67.8% of cases, whereas neurological infection accounted for 15.2%. Gastrointestinal infections accounted for 9.6% of cases, whereas bloodstream or other infections accounted for 7.3%. The median age of the overall cohort was 19 months (IQR, 4–77), and 201 patients (38.7%) had at least one comorbidity. Overall mortality was 6.2% (32/519), and the median time to death was 4 days (IQR, 1–9). Non-survivors were predominantly male (62.5%), with a median age of 39 months (IQR, 15–95). Survivors had a median stay of 8 days (IQR, 4–14), were 66.1% male, and had a median age of 17 months (IQR, 4–74). Fourteen patients (2.7%) experienced early death or required ECMO. Comparisons between the early death/ECMO group and the late death group are shown in Table 2. The PSS-based classification of organ dysfunction revealed that respiratory dysfunction was the most prevalent (89.0%), followed by coagulopathy (37.6%), cardiovascular dysfunction (26%), and neurological dysfunction (15.4%). Of these, neurological dysfunction had the strongest correlation with both the primary outcome (in-hospital mortality) and the secondary outcome (Early Death or ECMO) (21.3% and 11.3%, respectively), followed by cardiovascular dysfunction (18.5% and 9.6%), coagulopathy (14.4% and 7.2%), and respiratory dysfunction (6.5% and 2.6%) (Table 3).

**Table 1:** Baseline characteristics, scores of patients with confirmed or suspected infection at PICU admission.

Total	All (*n* = 519)	Survivors (*n* = 487)	Death(*n* = 32)	*p*-value
Male, n (%)	342(65.9)	322(66.1)	20(62.5)	0.68
Age, mon, median (IQR)	19(4–77)	17(4–74)	39(15–95)	0.01
Admssion source, n (%)				0.99
Emergency department	120(23.1)	113(23.2)	7(21.9)	
Inpatient	141(27.2)	135(27.7)	6(18.8)	
Other hospitals	258(49.7)	239(49.1)	19(59.4)	
Infection source, n (%)				0.03
Respiratory	352(67.8)	337(69.2)	15(46.9)	
Gastrointestinal	50(9.6)	44(9.0)	6(18.8)	
Neurological disease	79(15.2)	70(14.4)	9(28.1)	
bloodstream/Other	38(7.3)	36(7.4)	2(6.3)	
Complex Chronic Condition, n (%)				0.58
Yes	201(38.7)	189(38.8)	12(37.5)	
No	318(61.3)	298(61.2)	20(62.5)	
Organ dysfunction scores, median (IQR)				
PSS	2(1–3)	2(1–3)	7(4–9)	< 0.001
Phoenix-8	2(1–4)	2(1–4)	7(6–11)	< 0.001
SIRS	2(1–3)	2(1–3)	3(2–3)	< 0.001
pSOFA	3(2–6)	3(2–6)	11(8–13)	< 0.001
PELOD-2	3(0–5)	3(0–5)	10(6–15)	< 0.001
PRISM III	4(1–8)	4(1–8)	23(12–29)	< 0.001

**Table 2:** Baseline demographic and clinical characteristics of the early death/ECMO and late death groups.

Total	All (*n* = 32)	Early Death or ECMO (*n* = 14)	Late Deaths (*n* = 18)	*p*-value
Male, n (%)	20(62.5)	8(57.1)	12(66.7)	0.58
Age, Year, median (IQR)	39(15–95)	44(20–103)	38(7–91)	0.69
Admission source, n (%)				0.045
Emergency department	7(21.9)	6(42.9)	1(5.6)	
Inpatient	9(28.1)	3(21.4)	6(33.3)	
Other hospitals	16(50.0)	5(35.7)	11(61.1)	
Infection source, n (%)				0.19
Respiratory	15(46.9)	7(50.0)	8(44.4)	
Gastrointestinal	6(18.8)	1(7.1)	5(27.8)	
Neurological disease	9(28.1)	5(35.7)	4(28.6)	
bloodstream/Other	2(6.3)	1(7.1)	1(5.6)	
Complex Chronic Condition, n (%)				0.89
Yes	11(34.4)	5(35.7)	6(33.3)	
No	21(65.6)	9(64.3)	12(66.7)	
Organ dysfunction scores, median (IQR)				
PSS	7(4–9)	9(8–10)	6(2–7)	< 0.001
Phoenix-8	7(6–11)	11(9–13)	7(4–7)	< 0.01
SIRS	3(2–3)	3(2–3)	3(2–3)	0.87
pSOFA	11(8–13)	12(11–14)	10(6–13)	< 0.05
PELOD-2	10(6–15)	12(6–17)	10(5–14)	0.16
PRISM III	23(12–29)	27(23–34)	19(7–25)	< 0.01

**Table 3:** Distribution of Phoenix Sepsis Score organ system subscores and their association with in-hospital mortality and early death or ECMO.

Phoenix Sepsis Score Organ Systems	All(*n*,%)	In-Hospital Mortality	Early Death or ECMO
Total, n	519(100)	6.2%	2.7%
Respiratory			
Score ≥ 1 point	462(89.0)	6.5%	2.6%
0 point	57(11.0)	3.5%	3.5%
1 point	331(63.8)	3.9%	0.3%
2 points	105(20.2)	6.7%	1.9%
3 points	26(5.0)	38.5%	34.6%
Cardiovascular			
Score ≥ 1 point	135(26.0)	18.5%	9.6%
Low mean arterial pressure			
0 point	503(96.9)	5.4%	2.2%
1 point	14(2.7)	21.4%	7.1%
2 points	2(0.4)	100%	100%
High lactate			
0 point	478(92.1)	3.8%	0.8%
1 point	25(4.8)	28.0%	12.0%
2 points	16(3.1)	43.8%	43.8%
Vasoactive use			
0 point	398(76.7)	1.8%	0.3%
1 point	63(12.1)	4.8%	1.6%
2 points	58(11.2)	37.9%	20.7%
Coagulation			
Score ≥ 1 point	195(37.6)	14.4%	7.2%
Low platelets			
0 point	420(80.9)	2.9%	0.7%
1 point	99(19.1)	20.2%	11.1%
High INR			
0 point	387(74.6)	2.6%	0.8%
1 point	132(25.4)	16.7%	8.3%
Low fibrinogen			
0 point	511(98.5)	5.7%	2.2%
1 point	8(1.5)	37.5%	37.5%
High D-dimer			
0 point	415(80.0)	3.1%	0.7%
1 point	104(20.0)	18.3%	10.6%
Neurologic			
Score ≥ 1 point	80(15.4)	21.3%	11.3%
Glasgow Coma Scale			
0 point	439(84.6)	3.4%	1.1%
1 point	65(12.5)	6.2%	3.1%
Fixed pupils	15(2.9)	86.7%	46.7%

**Fig. 2:**
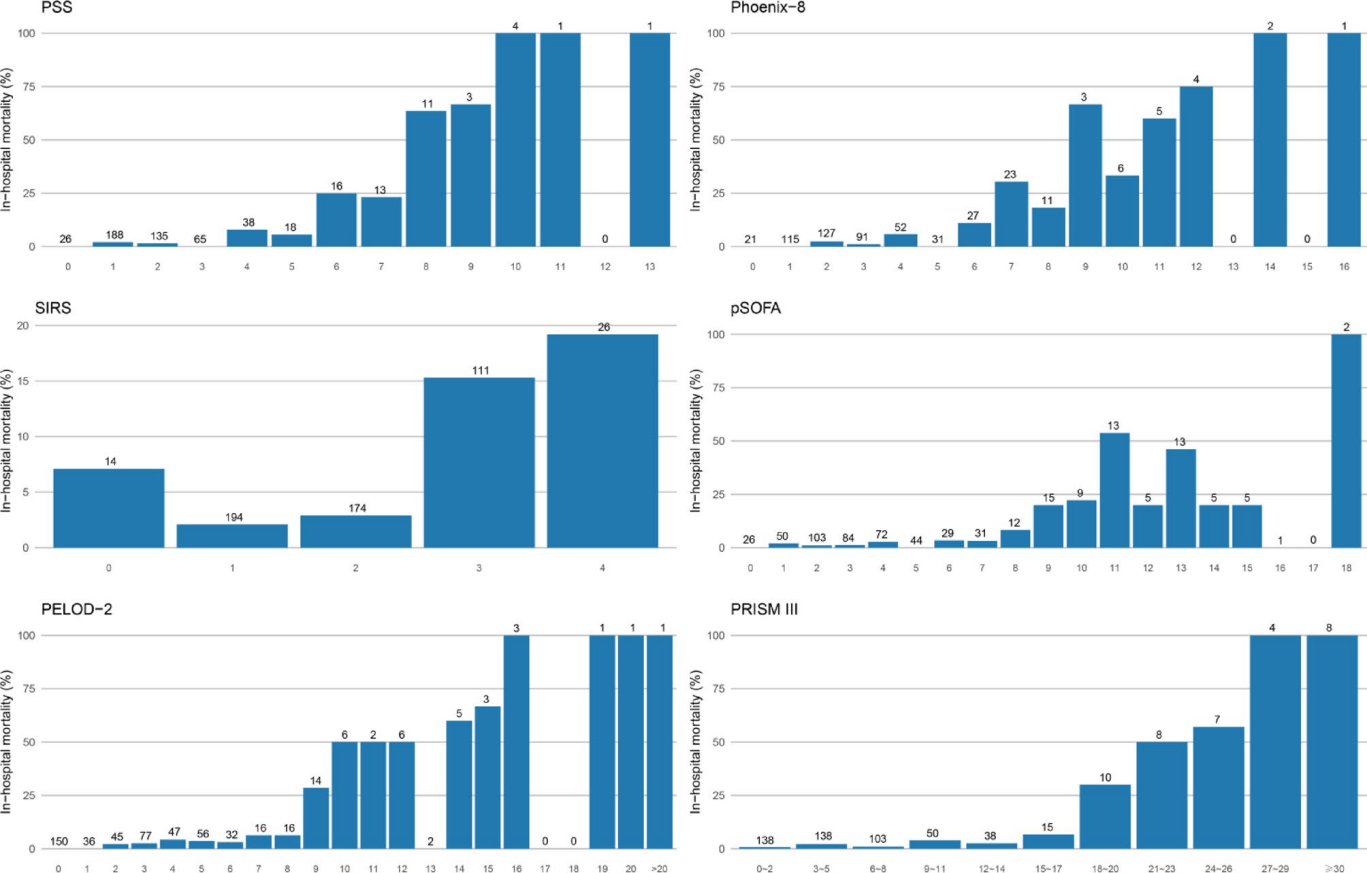
Mortality among patients, stratified by PSS, Phoenix-8, SIRS, pSOFA, PELOD-2, and PRISM III scores.

**Fig. 3:**
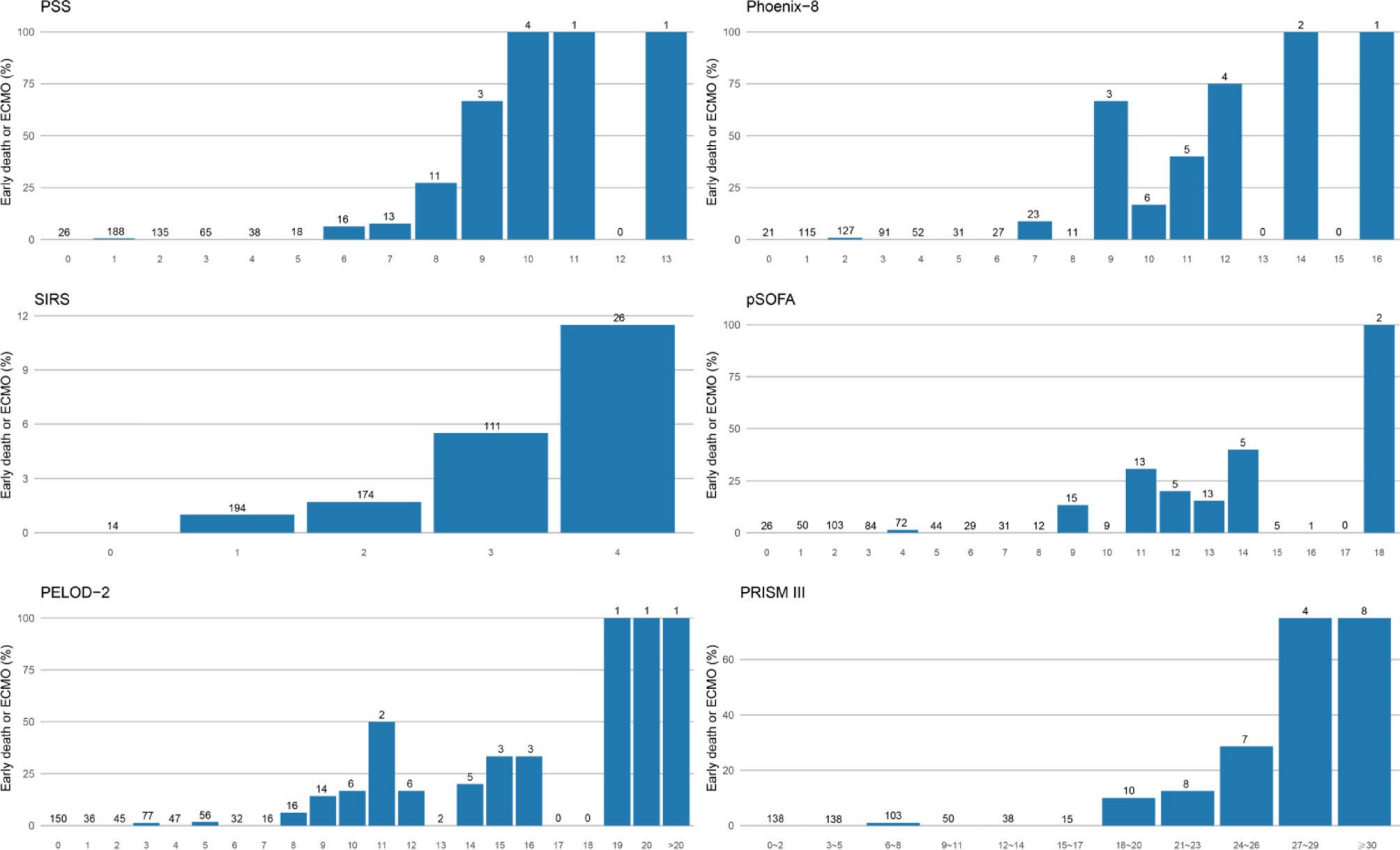
Early death or ECMO among patients, stratified by PSS, Phoenix-8, SIRS, pSOFA, PELOD-2, and PRISM III scores.

Among the 519 patients, non-survivors had significantly higher median scores across all scoring systems than survivors (Table 1). Among non-surviving patients, those who died early or required ECMO had significantly higher median PSS, Phoenix-8, pSOFA, and PRISM III scores than those who died later (Table 2). In risk stratification analyses, 305 patients (58.7%) had a PSS ≥ 2. Compared with patients with PSS < 2, mortality increased from 1.9% to 9.2% (absolute difference, 7.3%; 95% CI, 1.7%-12.2%; *p* < 0.001; Fig. 2), and the risk of the early death/ECMO increased from 0.5% to 4.3% (absolute difference, 3.8%; 95% CI, 1.3%-6.3%; *p* < 0.01; Fig. 3). A total of 443 patients (85.4%) had a pSOFA score ≥ 2. In this group, mortality increased from 1.3% to 7.0% (absolute difference, 5.7%; 95% CI, -2.1%-9.5%; *p* = 0.068; Fig. 2), while the incidence of the early death/ECMO increased from 0% to 3.2% (absolute difference, 3.2%; 95% CI, -2.9%- 5.2%; *p* = 0.24; Fig. 3). Similarly, 189 patients (36.4%) had a SIRS score ≥ 2, with mortality increasing from 2.4% to 8.7% (absolute difference, 6.3%; 95% CI, 0.5%-11.3%; *p* < 0.01; Fig. 2) and the early death/ECMO increasing from 1.0% to 3.9% (absolute difference, 2.9%; 95% CI, -1.2%-6.4%; *p* < 0.05; Fig. 3). A PELOD-2 score ≥ 2 was observed in 333 patients, among whom mortality increased from 0% to 9.6% (absolute difference, 9.6%; 95% CI, 4.9%-13.3%; *p* < 0.001; Fig. 2), and the early death/ECMO increased from 0% to 4.2% (absolute difference, 4.2%; 95% CI, 0.5%-6.9%; *p* < 0.01; Fig. 3).

**Fig. 4:**
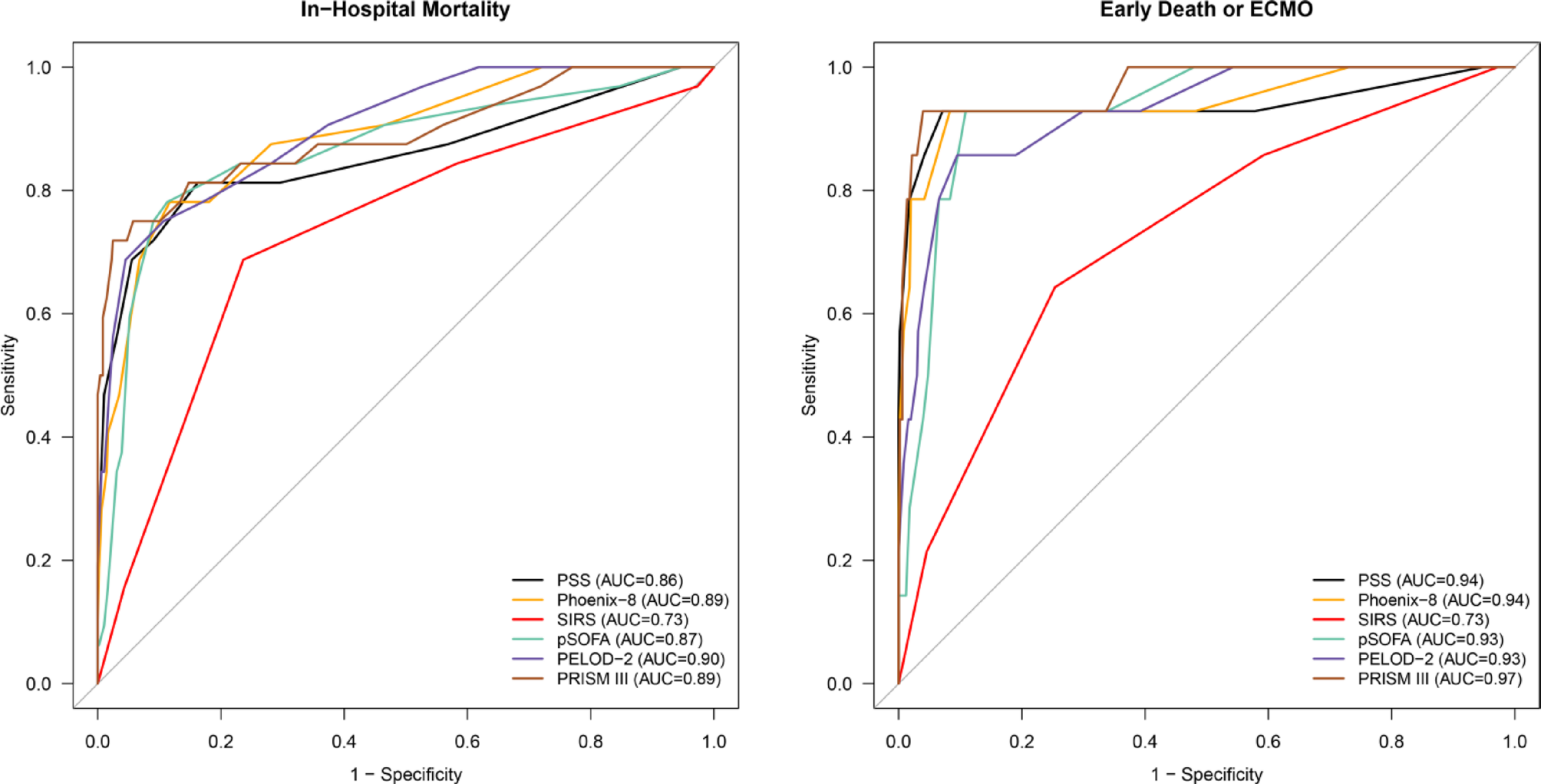
Discriminative performance of six scoring systems for in-hospital mortality and early death/ECMO, evaluated by AUC within 24 h of PICU admission in children with infection.

Table 4 summarizes the AUC of the scoring systems for the outcome, and Fig. 4 displays the corresponding ROC curves. For discrimination between survival and non-survival, PSS, Phoenix-8, pSOFA, PELOD-2, and PRISM III performed well (all AUC > 0.80), whereas SIRS was only acceptable (AUC = 0.73). The AUC of PSS was 0.86 (95% CI, 0.77–0.95). This value was higher than that of SIRS (AUC = 0.73; 95% CI, 0.63–0.83) (*p* < 0.05) and comparable to that of pSOFA (AUC = 0.87; 95% CI, 0.79–0.94), but lower than those of Phoenix-8, PELOD-2, and PRISM III, although no significant differences were observed (Table 5). The Hosmer–Lemeshow goodness-of-fit test with an adaptive binning strategy showed no evidence of significant miscalibration for any scoring system (Phoenix, *p* = 0.06; Phoenix-8, *p* = 0.39; SIRS, *p* = 0.15; pSOFA, *p* = 0.67; PELOD-2, *p* = 0.13; PRISM III, *p* = 0.14; Table 6). For the composite outcome distinguishing early death from late death, SIRS still showed only acceptable discrimination (AUC = 0.73; 95% CI, 0.59–0.87). In contrast, PSS, Phoenix-8, pSOFA, PELOD-2, and PRISM III showed strong discrimination. The AUCs were 0.94 (95% CI, 0.83–1.00), 0.94 (95% CI, 0.86–1.00), 0.93 (95% CI, 0.87–0.99), 0.93 (95% CI, 0.86–1.00), and 0.97 (95% CI, 0.92–1.00), respectively. Overall, the predictive ability of PSS was comparable to that of Phoenix-8. It was better than SIRS, pSOFA, and PELOD-2, but inferior to PRISM III.

**Table 4:** Discriminative performance of six scoring systems for in-hospital mortality and early death or ECMO.

Predictor	In-Hospital Mortality(6.2%)	Early Death or ECMO(2.7%)
PSS	0.86(0.77–0.95)	0.94(0.83-1.00)
Phoenix-8	0.89(0.82–0.95)	0.94(0.86-1.00)
SIRS	0.73(0.63–0.83)	0.73(0.59–0.87)
pSOFA	0.87(0.79–0.94)	0.93(0.87–0.99)
PELOD-2	0.90(0.85–0.96)	0.93(0.86-1.00)
PRISM III	0.89(0.82–0.97)	0.97(0.92-1.00)

**Table 5:** Comparison of the AUC for predicting in-hospital mortality between the PSS and other scoring systems, with pairwise difference testing.

Comparison	AUC1	AUC2	95% CI diff	*P* value
PSS vs. pSOFA	0.86	0.87	-0.052, 0.032	0.637
PSS vs. PELOD-2	0.86	0.90	-0.101, 0.009	0.100
PSS vs. PRISM III	0.86	0.89	-0.102, 0.033	0.316
PSS vs. Phoenix-8	0.86	0.89	-0.072, 0.008	0.116
PSS vs. SIRS	0.86	0.73	0.007, 0.244	0.037

**Table 6:** Calibration of six scoring systems assessed by the Hosmer–Lemeshow test in critically ill children.

Predicted probability	*n*	Non-survival		Survival		χ2	P*
		Observations	Estimates	Observations	Estimates		
PSS						5.47	0.06
0.000,0.009	214	4	1.682	210	212.32		
0.010,0.017	135	2	2.26	133	132.74		
0.018,0.033	65	0	2.15	65	62.85		
0.034,0.999	105	26	25.91	79	79.09		
Phoenix-8						3.04	0.39
0.000,0.008	136	0	0.92	136	135.08		
0.009,0.013	127	3	1.60	124	125.40		
0.014,0.022	91	1	1.99	90	89.00		
0.023,0.064	83	3	3.96	80	79.04		
0.065,0.999	82	25	23.53	57	58.47		
pSOFA						3.19	0.67
0.000,0.008	76	1	0.51	75	75.49		
0.009,0.011	103	1	1.12	102	101.88		
0.012,0.016	84	1	1.33	83	82.67		
0.017,0.023	72	2	1.64	70	70.36		
0.024,0.033	44	0	1.44	44	42.56		
0.034,0.095	72	3	4.58	69	67.42		
0.096,0.999	68	24	21.38	44	46.62		
SIRS						3.78	0.15
0.000,0.022	208	5	4.21	203	203.79		
0.023,0.051	174	5	8.74	169	165.26		
0.052,0.115	111	17	12.76	94	98.24		
0.116,0.999	26	5	6.29	21	19.71		
PLOED-2						2.34	0.13
0.000,0.008	186	0	0.93	186	185.07		
0.009,0.026	169	5	3.00	164	165.995		
0.027,0.999	164	27	28.06	137	135.94		
PRISM III						3.89	0.14
0.000,0.006	138	1	0.58	137	137.42		
0.007,0.011	138	3	1.09	135	136.91		
0.012,0.030	119	2	2.38	117	116.62		
0.031,0.999	124	26	27.95	98	96.05		

**Fig. 5:**
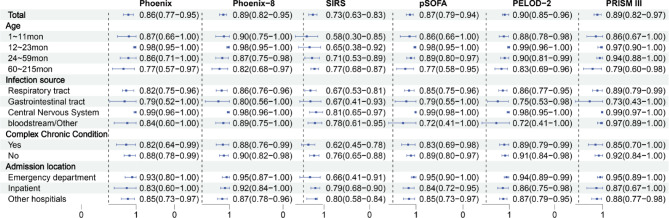
Forest plot of the AUC values for six scoring systems across all subgroups. The reference line indicates an AUC of 0.5.

As shown in Fig. 5, age stratification indicated that PSS, Phoenix-8, pSOFA, PELOD-2, and PRISM III exhibited relatively stable discrimination (AUC > 0.75), with superior performance in children younger than 5 years, whereas SIRS demonstrated substantial variability and poorer stability across age groups. Infection source stratification revealed higher discrimination in the neurological infection subgroup, in which PSS, Phoenix-8, pSOFA, PELOD-2, and PRISM III maintained strong discriminatory performance. Comorbidity stratification was associated with better discrimination across all scoring systems in patients without comorbidities. Admission source stratification demonstrated superior discrimination of PSS, Phoenix-8, pSOFA, PELOD-2, and PRISM III among patients admitted from the emergency department, whereas SIRS performed relatively better among ward admissions and inter-hospital transfers.

## Discussion

In this prospective cohort study conducted in low- and middle-income countries, we evaluated the ability of PSS, Phoenix-8, SIRS, pSOFA, PELOD-2, and PRISM III in predicting in-hospital mortality and the composite outcome of early death or ECMO among children admitted to the PICU with confirmed or suspected infection. Compared with SIRS, PSS demonstrated higher predictive accuracy for both outcomes and showed performance comparable to pSOFA. Phoenix-8, PELOD-2, and PRISM III exhibited better discrimination for in-hospital mortality. For the prediction of early death or ECMO, reflecting rapid disease progression, PSS performed similarly to pSOFA, Phoenix-8, and PELOD-2, although it remained slightly inferior to PRISM III.

The observed differences in predictive performance among the evaluated scoring systems likely reflect differences in their underlying design and clinical focus rather than simple variations in overall quality. SIRS is mainly applied to the early detection of systemic inflammatory responses. pSOFA and PSS, by contrast, focus on sepsis-related phenotypes and assess the presence and severity of organ dysfunction in the context of infection. PELOD-2 specifically quantifies the severity of multiple organ dysfunction, while PRISM III combines physiological and laboratory indicators to estimate overall disease severity and the risk of mortality. Accordingly, for the prediction of in-hospital mortality, severity-oriented scoring systems, including PELOD-2 and PRISM III, demonstrated greater discrimination.

Since the first pediatric sepsis consensus definition was introduced in 2005 and the Sepsis-3 criteria were subsequently established in 2016, multiple diagnostic standards and scoring systems have been widely applied in pediatric research and clinical practice^5,8,18–23^. Large PICU cohort studies from Australia, New Zealand, and the United States, as well as studies conducted in China, have consistently shown that pSOFA performs well in predicting outcomes among children with sepsis^8,20,22,23^. Our study also showed that pSOFA had good discriminative ability and good calibration, which provides further support for its broad use in pediatric patients with infection and sepsis. In 2024, the Pediatric Sepsis Definition Task Force released updated diagnostic criteria for pediatric sepsis and septic shock, leading to increasing use of the PSS in outcome assessment. Previous validation studies of the PSS have shown varying predictive performance across different regions and cohorts. For instance, an emergency department cohort in Australia and New Zealand reported an AUC of 0.75 for predicting in-hospital mortality, while a U.S. PICU cohort showed an AUC of 0.82, and a European PICU cohort demonstrated an AUC of 0.88. A cohort from Korea reported an AUC of 0.85^13–16^. By comparison, a prior single-center Chinese study, using a PSS threshold of ≥ 2 to predict in-hospital mortality, reported an AUC of 0.81^17^. It should be noted that, except for the Chinese single-center study, all other studies used continuous PSS scoring. This could influence the comparability of AUC results between studies. Compared to the emergency department setting, pediatric patients in the PICU tend to have more severe baseline conditions and a higher incidence of organ dysfunction. Under these conditions, organ dysfunction–oriented scoring systems, such as PSS and pSOFA, may better capture the true risk gradient than SIRS. This context also helps explain the better performance of PSS in PICU cohorts compared with earlier emergency department studies, suggesting that it may be more applicable in critical care settings.

PELOD-2 is a well-recognized scoring system for quantifying organ dysfunction in pediatric patients and has been evaluated in numerous studies, particularly in the prognostic assessment of children with suspected infection or sepsis. Its ability to discriminate in-hospital mortality has been consistently reported^22,24–30^. In our cohort, PELOD-2 showed predictive performance for in-hospital mortality comparable to that reported in prior studies, with acceptable calibration. Compared with PSS, SIRS, and pSOFA, PELOD-2 allows for repeated daily assessment, enabling dynamic evaluation of the progression of organ dysfunction and providing more comprehensive information for ongoing severity assessment. However, in clinical practice in low- and middle-income countries, the routine implementation of daily scoring is often challenging. Further prospective studies are therefore needed to evaluate the feasibility and prognostic value of serial PELOD-2 assessments in these settings.

PRISM III has long been recognized as a reliable tool for assessing the severity of pediatric critical illness and has consistently shown good prognostic accuracy in various studies^22,25,28,31,32^. In the present study, we confirmed its high discriminative performance in predicting both in-hospital mortality and the composite outcome of early death or ECMO among children with infections. The Phoenix-8 score, an extension of the PSS, has also shown robust predictive performance for adverse outcomes in pediatric infections. Its discrimination was comparable to that of PRISM III, a well-validated severity scoring system. These findings indicate that Phoenix-8 may have potential applicability beyond infectious conditions. However, its use in non-infectious pediatric populations requires further investigation in future studies.

We performed subgroup analyses focusing on several key strata. First, better performance was observed for all tools among children without complex chronic conditions (CCC). This may indicate that, in this subgroup, acute sepsis-related organ dysfunction contributes more directly to outcome variation. Second, PSS, Phoenix-8, pSOFA, PELOD-2, and PRISM III showed higher predictive performance in emergency admissions than in ward admissions or inter-hospital transfers. Pre-PICU treatment is common in ward admissions and transfers, including vasoactive support, antibiotics, and fluid resuscitation. Reliance on data collected at PICU admission alone may therefore lead to underestimation of disease severity. Conversely, SIRS showed better performance in ward admissions and transfers than in emergency admissions. This pattern may be explained by the time-dependent nature of inflammatory manifestations captured by SIRS. In emergency admissions, inflammation may not yet have fully evolved, which may limit mortality discrimination. Finally, across infection-source subgroups, relatively better performance was observed in central nervous system infections, potentially because the proportion of CCC was lower in this subgroup in our cohort.

This study has several limitations. First, separate adjustments were not performed for children with chronic organ dysfunction, such as those with immunosuppression or thrombocytopenia related to malignancy, during the scoring process. Consequently, these patients may have been more likely to meet diagnostic criteria for sepsis when infection was suspected. Further studies focusing on these specific populations are warranted. Second, although the cohort was derived from a low- and middle-income country, the study was conducted at a regional pediatric referral center with a high proportion of children with complex chronic conditions, which may have introduced selection bias. Finally, while the prospective design helped reduce information bias, the single-center nature of the study, along with the limited sample size and population representativeness, remains an important limitation. Future multicenter studies with larger sample sizes are required to corroborate these results.

In conclusion, we evaluated the ability of several risk scoring systems in predicting outcomes for PICU patients with infections. PSS demonstrated good discriminatory ability in identifying high-risk patients for mortality, outperforming SIRS and showing comparable performance to pSOFA. Although PELOD-2 showed higher accuracy in predicting adverse outcomes, PSS maintained high predictive performance while offering the advantages of simplified parameters and good usability. Therefore, PSS provides a practical option for early identification of in-hospital adverse outcomes in PICU patients with infections.

## Data Availability

The data utilized in this study are available from the corresponding author upon request.
